# Effects of Melatonin in Women with Polycystic Ovary Syndrome Undergoing ART: A Systematic Review

**DOI:** 10.3390/antiox15070896

**Published:** 2026-07-20

**Authors:** Sapna Jaiswar, Mahadev Bhise, Pallavi Shukla

**Affiliations:** 1 Department of Molecular Endocrinology, Indian Council of Medical Research-National Institute for Research on Women’s Health (ICMR-NIRWoH), (Formerly, Indian Council of Medical Research-National Institute for Research in Reproductive and Child Health (ICMR-NIRRCH)), Mumbai 400012, India; 2 Department of Biostatistics, Indian Council of Medical Research-National Institute for Research on Women’s Health (ICMR-NIRWoH), (Formerly, ICMR–National Institute for Research in Reproductive and Child Health (ICMR-NIRRCH)), Mumbai 400012, India; 3 Academy of Scientific and Innovative Research, Ghaziabad 201002, India

**Keywords:** PCOS, melatonin, ART, oocyte quality, pregnancy rate

## Abstract

Polycystic ovary syndrome (PCOS), newly named as Polyendocrine Metabolic Ovarian Syndrome (PMOS) is the most common endocrinopathy affecting women of reproductive age, which often leads to infertility due to anovulation and poor oocyte quality. Poor oocyte quality is a major reason for women’s infertility in humans and a barrier to efficient assisted reproduction technology (ART) treatment. Supplementation of melatonin, a free radical scavenger, has been reported to show satisfactory results in women undergoing ART. This review investigates whether the endogenous melatonin or supplementation of melatonin impact on oocyte/embryo quality and pregnancy rate in PCOS patients undergoing ART treatment. Articles published between 2010 and 2025 were reviewed to perform a comprehensive search on observational studies and clinical trials that evaluated the use of melatonin in PCOS patients undergoing in vitro fertilization (IVF) or intrauterine insemination (IUI) or intracytoplasmic sperm injection (ICSI). The databases searched included PubMed, Google Scholar, Science Direct, and Cochrane Library. This systematic review indicates that the group that received melatonin had significantly higher clinical pregnancy probabilities than the control group. Similarly, the melatonin administration significantly enhanced the endometrium thickness (*p* < 0.001) and the chemical pregnancy rate (30% versus 18%, *p* = 0.012). Additionally, in comparison to the control group that received metformin alone, the group that received melatonin (3 mg) significantly increased the proportion of top-quality embryos (40.3% vs. 29.9%; *p* < 0.001). According to this systematic review, melatonin significantly improves endometrial thickness, mature oocytes, high-quality embryos, and pregnancy rates, all of which improve ART outcomes in PCOS patients. Because of its safety, affordability, and efficacy, it is a useful supplement to ART procedures. The most popular and well-tolerated dosage is still 3 mg per day, despite variations in research.

## 1. Introduction

Polycystic ovary syndrome (PCOS) or PMOS is the most prevalent disorder causing infertility in women of reproductive age. The prevalence of PCOS ranges from 5% to 15%, depending on the diagnostic standards used [1]. Women with PCOS frequently seek treatment for irregular menstrual cycles and infertility. These symptoms are caused by chronic oligo/anovulation [2]. In addition, altered circadian rhythm and sleep disturbances are significant issues among patients with PCOS [3]. The cause of PCOS is yet unknown. It may involve a combination of environmental risk factors, such as poor lifestyle choices and an unhealthy diet, along with genetic and epigenetic factors [4]. The variety of ways that PCOS manifests itself indicates that a single cause is unlikely [5]. Since PCOS is the primary cause of infertility in women, other etiologies of infertility, such as other endocrine diseases, should be ruled out before a definitive diagnosis is made [6]. Additionally, miscarriage and pregnancy issues, such as gestational diabetes, may be more likely in women with PCOS [7]. Infertility is defined as the failure to conceive within a year of unprotected sexual activity. In both developing and emerging nations, infertility is becoming increasingly common as both a social and a medical problem [8]. Unfortunately, in ART, the main reason for unsuccessful fertilization is low-quality oocytes. Over 60% of IVF cycles do not result in pregnancy [9]. To address these fertility issues, many couples use ART. These include ICSI, IUI, IVF, and ovulation stimulation [10]. Thus, there is a growing interest in finding adjunctive treatments that could enhance IVF success rates in women with PCOS.

Melatonin is the main hormone released by the pineal gland [11]. The biological clock located in the hypothalamus controls the circadian pattern of its production. Among the primary roles of melatonin’s rhythmic production in the blood are immunological response, circadian rhythm modulation, and sleep regulation [12]. Melatonin’s primary roles are as an antioxidant and a free radical scavenger [13]. Another biologically significant hormone used in PCOS treatment is Metformin. Metformin reduces circulating insulin and glucose via blocking hepatic glucose synthesis, reducing lipid synthesis, increasing fatty acid oxidation, and inhibiting gluconeogenesis [14]. Although metformin co-treatment may improve ART outcomes, it has its own limitations. However, in women with PCOS undergoing ART, there is a need to explore new treatments to improve ART outcomes that require understanding PCOS pathophysiology at the ovarian level.

The causes of PCOS are not entirely understood, although oxidative stress (OS) is among several environmental and epigenetic factors that may play a significant role in the development of the disorder [15]. OS in the cell occurs when the production of reactive oxygen species (ROS) and other radical species surpasses the antioxidants’ scavenging ability because of the high generation of ROS and/or insufficient consumption or increasing use of antioxidants [16]. Further, as mitochondria produce most of the ROS that cause OS, mitochondrial dysfunction may also play a key role in the pathophysiology of PCOS [17]. Genetic alterations in mitochondrial genes and nuclear-related mitochondrial genes may result in OS and impaired energy metabolism [18]. Mitochondrial dysfunction can lead to an increase in ROS, which induces OS, which can play a major role in causing PCOS. Mitochondrial DNA alterations are mainly single nucleotide polymorphisms [17] unlike large deletions, as in the case of other disorders [19]. Excess ROS in follicular fluid is known to cause granulosa cells and oocytes to undergo apoptosis, which lowers the quality of oocytes. Intracellular antioxidant systems like glutathione (GSH) can prevent these negative effects of ROS; therefore, maintaining a balance between ROS generation and detoxification is crucial for oocyte quality [8]. Moreover, there is a reduction in antioxidant concentration in the follicular fluids of PCOS patients, which causes the ovary’s luteal and follicular phases not to cycle properly. All these factors are the major reasons for poor oocyte quality. Melatonin has been shown to regulate several physiological functions, including mitochondrial homeostasis, apoptosis and autophagy, endoplasmic reticulum stress response, and control of circadian rhythms. By scavenging free radicals, cells are also protected from oxidative stress by melatonin and its metabolites [20]. According to recent studies, Melatonin shields cells from OS by stimulating antioxidant enzymes and acting as a free radical scavenger. As a result, melatonin administration can rescue oocytes from OS, perhaps resulting in improved ART outcome in IVF patients [21].

It has recently been demonstrated that melatonin, which is usually produced by the body, can be a potential predictor of the quality of oocytes and the success of ART [22]. Its high concentrations positively correlate with oocytes of satisfactory quality. It helps in reducing insulin resistance, which is the primary cause of metabolic dysfunctions occurring in PCOS/PMOS [23].

This systematic review aimed to synthesize evidence from clinical trials and observational studies to evaluate whether melatonin supplementation improves oocyte and embryo quality, endometrial thickness, and pregnancy outcomes in women with PCOS/PMOS undergoing ART treatment.

## 2. Methods

### 2.1. Literature Search

The systematic review was registered in PROSPERO (CRD420251111556). Two reviewers independently searched the literature from January 2010 to December 2025 using Google Scholar, Science Direct, Cochrane Library, and PubMed. Patients were those undergoing IVF, IUI, or ICSI treatment and diagnosed with PCOS. Interventions included melatonin supplementation administered either orally or in-vitro. Treatments devoid of melatonin or combined with a placebo served as comparators. Primary outcomes included oocyte and embryo quality and pregnancy rates. The study design was randomized controlled trials. The key search terms included, but were not limited to, “Melatonin” OR “Melatonina” OR “Melatonine” OR “5-methoxy-N-acetyl tryptamine” AND “Polycystic ovary syndrome”, “PCOS” OR “Polycystic ovary disease” AND “In Vitro Fertilization” OR “Assisted Reproductive Technology”. The search terms and methods are provided in detail in Supplemental Table S1.

### 2.2. Inclusion Criteria

**Figure 1:**
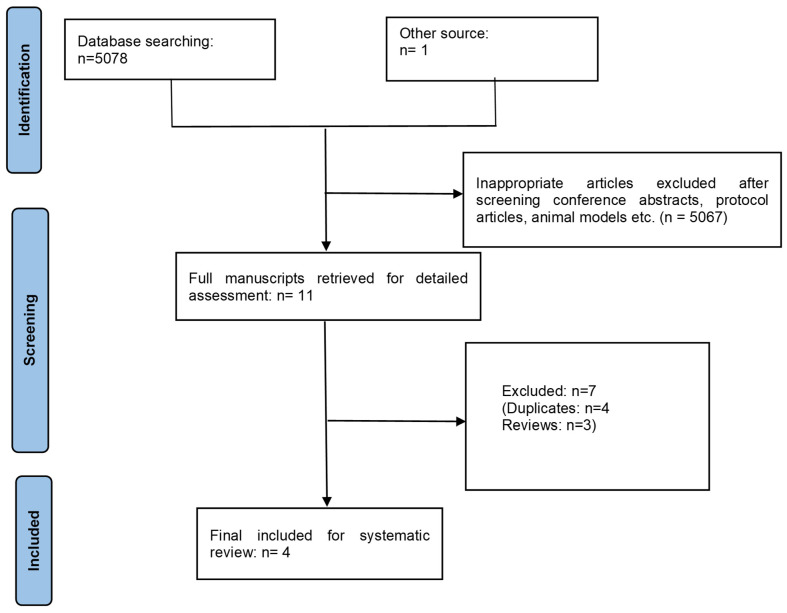
PRISMA flow diagram for included studies in the systematic review.

We considered observational studies and randomized clinical trials that examined the effects of melatonin in women with PCOS undergoing ART, including both in vitro supplementation for oocyte or embryo culture and in vivo treatment. Excluded studies were those not written in English or that used animal models. Additionally, case reports, reviews, conference abstracts and study protocols were excluded (Figure 1).

### 2.3. Study Selection

Titles and abstracts were independently assessed by two reviewers using the pre-established inclusion criteria. Studies were retained for full-text review if initial screening indicated potential eligibility. A third reviewer was consulted to settle any disagreements during the selection procedure.

### 2.4. Data Extraction

Two reviewers independently extracted data from the included studies, with disagreements resolved by a third reviewer. In cases of multiple publications from the same study, the primary report was used, supplemented by additional details from related articles when necessary. Extracted data included sample size, publication year, study duration, country, eligibility criteria, diagnostic criteria, IVF protocols, outcome definitions, and relevant outcome data. ART outcomes such as oocyte and embryo quality, endometrial thickness, pregnancy rate, implantation rates, fertilization rates, and live birth rate were examined in each study. Data from each treatment group were combined and treated as a single intervention group in studies that included multiple melatonin dosage groups.

### 2.5. Risk of Bias

The quality of the included studies was assessed independently by two reviewers using the RoB2.0 tool based on the Cochrane Collaboration’s criteria (version 5.1.0, available at www.cochrane-handbook.org, accessed on 7 August 2025). Studies were categorically classified as having a low, high, or unclear risk of bias (Table 1). Out of the four studies, two were judged to have a moderate overall risk of bias, demonstrating strong methodological rigor in randomization, intervention fidelity, outcome assessment, and result reporting. One study showed some concerns, mainly due to insufficient details on blinding or incomplete reporting of outcomes; however, these studies maintained acceptable standards in most domains and contributed meaningfully to the review. One study was rated as having a high risk of bias across multiple domains, largely because it was observational in nature and lacked randomization and blinding procedures; its findings should therefore be interpreted with caution. Despite these limitations, the overall quality of evidence was considered acceptable, and the findings were regarded as reliable for drawing preliminary conclusions about the role of melatonin in improving IVF/IUI outcomes in women with PCOS.

This study was primarily conducted as a systematic review. Due to heterogeneity in study design, interventions, and outcome reporting, a full meta-analysis was not feasible.

## 3. Results

### 3.1. Characteristics of Included Studies

The review process was conducted in accordance with PRISMA guidelines, with an initial record of 5079 articles (Figure 1). Ultimately, four studies, including randomized, observational, and clinical trials, were included for analysis [3,15,21,24]. The characteristics of the included studies are displayed in Table 2 and Table 3. The included studies were published between 2010 and 2023. Sample sizes ranged from 35 to 320 women with PCOS, and all four studies focused exclusively on women with PCOS. Two studies concentrated on women undergoing IVF, one included women undergoing IUI and one included participants undergoing either IVF or ICSI. Across these trials, 3 mg/day of melatonin was the most commonly utilized dosage.

### 3.2. Impact of Melatonin on Oocyte/Embryo Number/Quality and Fertilization Rate

According to Li et al., women with PCOS had significantly lower melatonin concentrations in their follicular fluid than normal control groups (*p* < 0.045), and lower melatonin levels were linked to lower rates of top embryo quality on day 3 and top blastocyst quality on day 5/6 [3]. However, the number of oocytes retrieved and the IVF rate were higher in the PCOS group compared to the control group [3]. According to Pilehvari et al. (2023), compared to the control group receiving metformin alone, the group receiving melatonin (3 mg) in addition to metformin showed a significantly higher proportion of top-quality embryos (40.3% vs. 29.9%; *p* < 0.001), a significantly greater total number of oocytes retrieved (*p* < 0.001), and a higher proportion of metaphase (MII) oocytes (69.9% vs. 57.9%) [15]. Mokhtari et al. (2019) identified an increased number of mature follicles in the melatonin-treated group but did not investigate the impact on oocyte/embryo quality [21]. However, Kim et al. found that oocyte maturation, fertilization, cleavage, and embryonic development in the melatonin-treated group were similar to those in the control group [24].

### 3.3. Impact of Melatonin on Endometrial Thickness and Pregnancy Outcomes

According to Pilehvari et al. (2023), the group that received melatonin as a co-treatment had 1.8 times the clinical pregnancy odds of the control group that received only metformin (*p* = 0.039) [15]. Furthermore, the melatonin treatment group had a lower rate of spontaneous abortion than the control group, although the difference was not statistically significant (*p* = 0.520). Similarly, Mokhtari et al. (2019) found that, compared to the control group, melatonin administration significantly enhanced the endometrial thickness (9.2% vs. 8.5% *p* < 0.001), chemical pregnancy rate (30% vs. 18%, *p* = 0.012) and clinical pregnancy rate (26% vs. 15%, *p* = 0.013) [21]. Also, the addition of melatonin to treatment cycles in PCOS patients significantly improved follicle size during IUI cycles (*p* = 0.002) [21]. Kim et al. (2013) [24] found that when melatonin was added to the in vitro culture medium (IVM) in both the non-stimulation group and the HCG priming group, the implantation rate was higher in the melatonin group (*p* < 0.05) and pregnancy rates were slightly improved, though not significantly different from controls. In phase II, HCG priming with melatonin supplementation in the IVM used during the IVM/IVF-embryo transfer program (phase-II study), successfully led to a clinical pregnancy rate of >40% in PCOS participants. Li and group also found no significant difference in embryo implantation rate, clinical pregnancy rate, early miscarriage rate, or live birth rate between the PCOS group with lower follicular fluid melatonin concentrations and the non-PCOS group after participant follow-up [3]. Due to heterogeneity in study design, interventions, and outcome reporting, a full meta-analysis was not feasible. 

## 4. Discussion

This systematic review includes clinical trials and experimental studies for quantitative analysis, demonstrating that melatonin supplementation, either by itself or in conjunction with medications such as metformin or myo-inositol, significantly raises clinical pregnancy rates. This effect is primarily due to an improvement in the quality of the oocytes and embryos.

A highly prevalent condition in women associated with infertility, PCOS is characterized by hyperandrogenism, polycystic ovarian morphology (PCOM; an excess of preantral follicles in the ovaries), and ovulatory dysfunction, including menstrual dysfunction [25]. Additionally, higher rates of sleep issues, anxiety, and depression are observed among PCOS patients [26]. PCOS is mostly associated with anovulatory infertility, which is mainly caused by increased OS within the follicular environment, resulting in impaired oocyte development. An imbalance between pro-oxidants and antioxidants causes OS. A decrease in antioxidant defense systems or elevated levels of ROS and/or reactive nitrogen species (RNS) can alter this ratio [27]. OS is considered a factor contributing to low oocyte quality [28]. Furthermore, elevated levels of ROS in the follicular fluid of infertile women are negatively correlated with both oocyte maturation and embryo quality [29]. ROS production and elimination by enzymatic and non-enzymatic antioxidants are critical for preserving oocyte quality and, consequently, a female’s reproductive health [30].

**Figure 2:**
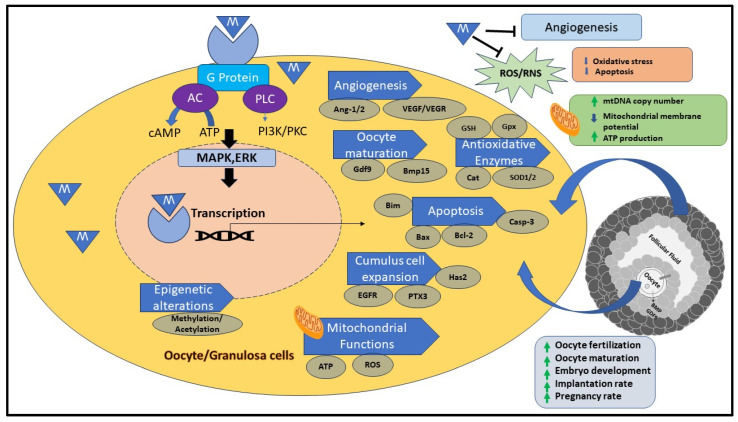
Impact of melatonin mediated by receptor and receptor independent mechanism. Melatonin acts by altering gene expression, modulating epigenetic changes and inhibit ROS/RNS directly leading to improved mitochondrial functions and ART outcome. The arrows (⊣) shows inhibition, upward arrow (↑) and downward arrow (↓) show increase and decrease in the process respectively.

Melatonin is an indoleamine released by all cells, including those in the pineal gland [31]. The synthesis of melatonin begins with tryptophan. Tryptophan hydroxylase transforms it into 5-hydroxytryptophan, which arylalkylamine N-acetyltransferase (AANAT) then converts to acetylated serotonin, specifically N-acetylserotonin (NAS), which acetylserotonin O-methyltransferase (ASMT) then converts to melatonin. The latter is also referred to as hydroxy-indole-O-methyltransferase (HIOMT) [32]. The direct function of melatonin in the ovaries has been the primary focus of research on its role in the reproductive process [33]. Melatonin has been shown to control several physiological functions, such as mitochondrial homeostasis, apoptosis and autophagy, endoplasmic reticulum stress response, and circadian rhythms. By scavenging free radicals, melatonin and its metabolites also protect cells from OS [20]. According to recent studies, melatonin shields cells from OS by stimulating antioxidant enzymes and acts as a free radical scavenger. It has the capacity to remove both ROS and RNS (Figure 2). It particularly detoxifies the hydroxyl radical (•OH), which is highly reactive and toxic. Further, melatonin is metabolized to produce metabolites including cyclic 3-hydroxmelatonin (C3OHM), N1-acetyl-N2-formyl-5-methoxykynuramine (AFMK) and N1-acetyl-5-methoxykynuramine (AMK), which possess potent antioxidative actions and regulate various cellular functions [31]. Melatonin protects granulosa cells from ROS by reducing OS of cellular components including the nucleus, mitochondria, and cell membranes (Figure 2). Indirect effects of melatonin via nuclear receptors such as, retinoid-related orphan receptor α (RORα) and/or membrane receptors like melatonin membrane receptors type 1 and type 2 (MT1, MT2) are considered crucial for oocyte maturation and embryonic development. MT1 and MT2 are located in oocytes and cumulus granulosa cells (cGCs) [31]. Both receptors are generally coupled to proteins, causing a reduction in intracellular cAMP and regulating signaling pathways such as protein kinase C (PKC), mitogen-activated protein kinase (MAPK), and phosphoinositide 3-kinase (PI3K)/protein kinase B (Akt) (Figure 2) [31]. Melatonin regulates the expression of genes related to oocyte maturation and embryonic development, including genes involved in cumulus cell expansion, prostaglandin-endoperoxide synthase 1 (*PTGS1*), prostaglandin-endoperoxide synthase 2 (PTGS2), and hyaluronan synthase 2 (HAS2), as well as sonic hedgehog (*SHH*) signaling-related genes and proteins in cumulus cells, including SHH, patched 1 (*PTCH1*), smoothened (*SMO*), and GLI family zinc finger 1 (*GLI1*). Additionally, melatonin influences apoptosis by attenuating the transcript level of caspase-3 and increasing the expression of B-cell lymphoma 2 (BCL-2), X-linked inhibitor of apoptosis protein (XIAP), catalase (CAT), and heat shock protein 70 (HSP70). Further, melatonin is also responsible for gene expression via epigenetic modifications such as DNA methylation and histone acetylation. Melatonin is produced in the mitochondria and enhances mtDNA copy number, membrane potential, and ATP production in oocytes and accelerates IVF embryo development [34]. Additionally, melatonin downregulates COX-2 protein levels and improves the activity of antioxidant enzymes through activation of the Nrf2 signaling pathway.

Hormonal abnormalities, insulin resistance, chronic inflammation, OS, and mitochondrial dysfunction are the hallmarks of PCOS. Melatonin regulates the endocrine system across the lifespan of female and influences ovulation. Its level declines with age, which may contribute to reduced fertility. Excess estrogen can lead to progesterone resistance, contributing to PCOS. Melatonin inhibits estrogen production in pre-ovulatory follicles through MAPK signaling, suppressing aromatase activity, and reducing estrogen receptor expression in ovarian and uterine tissues. Melatonin functions as an anti-angiogenic agent, as it also inhibits the production of angiopietin-1 and -2 (Ang-1/2), vascular endothelial growth factor (VEGF), and vascular endothelial growth factor receptor (VEGFR) during hypoxia, hence influencing neovascularization and angiogenesis, which are altered in PCOS [35]. In placental cells, melatonin is constantly produced and helps cytotrophoblasts survive while lowering ROS and excessive angiogenesis. As a result, melatonin administration can provide protection from OS to oocytes and a favorable environment for the endometrium as well as the placenta, leading to positive ART outcome in patients [21].

Melatonin levels were higher in the fluid of the large follicles of patients undergoing IVF embryo transfer than in the fluid of the small follicles [9]. It has recently been demonstrated that melatonin and myo-inositol, which are usually produced by the body, are effective predictors of oocyte quality and the success rate of ART: in fact, their high concentrations positively correlate with oocytes of satisfactory quality. Significance of OS in ART has grown in recent years, especially concerning ICSI and IVF methods [36].

According to a previous systematic review and meta-analysis, melatonin therapy significantly increases the number of oocytes recovered and dramatically increases the clinical pregnancy rate. Additionally, there is no difference in the miscarriage rate between the control group and the melatonin treatment group. Women taking melatonin have been known to experience several obstetric difficulties. Since only one patient is reported for each obstetric issue, this does not necessarily imply that melatonin medication raises the incidence of obstetric difficulties [20]. Another earlier prospective cohort study found that, while the difference in the number of recovered oocytes between the two IVF cycles was not noticeable, the combination of melatonin and myo-inositol treatment significantly increased the number of mature oocytes in women who were unable to conceive in prior cycles due to poor oocyte quality [9]. Additionally, implantation and pregnancy rates were positively impacted by a greater quantity of high-quality embryos [9]. These findings imply that myo-inositol and melatonin supplementation affect the general quality of the oocyte pool in addition to boosting the quantity of mature oocytes.

Studies included in our review [15,21] observed enhancement in oocyte maturation and embryo quality after melatonin treatment, supporting the hypothesis that melatonin acts as an antioxidant and enhances reproductive potential. For example, Pilehvari et al. [15] showed that melatonin therapy increased the quantity of high-quality embryos and mature oocytes relative to the control group. Other studies observed higher clinical pregnancy rates and implantation rates when supplemented with melatonin [21], and an increase in chemical pregnancy rate in PCOS women undergoing IUI, when melatonin was given as a treatment protocol. Similarly, Pilehvari and co-workers [15] noted a 1.8-fold increase in clinical pregnancy odds in the intervention group treated with melatonin. All these improvements were statistically significant for pregnancy, but evidence about live birth outcomes is still scarce.

Combination therapy, which is typically administered to increase the success rate of ART in PCOS patients, appears to augment the benefits of melatonin. Pilehvari and group [15] showed that the addition of metformin along with melatonin enhances the number of oocytes and improves embryo quality. The effectiveness, low cost, and safety profile of melatonin make it an attractive adjuvant in IVF protocol for PCOS patients. Even though much research has varied dosages and durations, 3 mg/day remains the most commonly utilized and well-tolerated dose. Crucially, none of the included trials showed any notable side effects, indicating that melatonin is safe for use in women of reproductive age.

Melatonin plays several roles in pregnancy, such as supporting placental function, fetal development, and influencing labor and neonatal health. Unlike findings from most clinical studies to date, evidence suggests that melatonin use during pregnancy and breastfeeding is probably safe in humans [37]. During pregnancy, melatonin levels increase progressively, peaking in the third trimester and during labor, before dropping sharply after delivery. In high-risk pregnancies, melatonin appears to have protective roles. Reduced melatonin levels have been linked to conditions such as gestational diabetes, preeclampsia, and intrauterine growth restriction (IUGR) [38]. In preterm infants, melatonin levels are very low because they miss the late-pregnancy maternal supply. In newborns, melatonin shows potential in treating conditions linked to OS, such as hypoxic–ischemic encephalopathy [38]. Animal studies demonstrate its ability to reduce brain infarction and individual cortical lesion sizes, as well as increase the number of surviving neurons, through anti-inflammatory and anti-apoptotic mechanisms [38].

Despite the encouraging results, several limitations should be taken into account. First, direct comparisons are limited by differences in study designs, intervention procedures, and outcome reporting. Second, particularly for live birth data, a large number of the included studies had short follow-up periods and small sample sizes. Third, some studies failed to stratify outcomes by PCOS phenotype, which may have an impact on treatment response. Additionally, single-center studies provide the majority of the evidence, which may restrict generalizability. However, given its safety and low cost, melatonin may be considered as an adjunctive therapy during ART for PCOS patients, but further large-scale randomized controlled trials (RCTs) are needed to confirm its effect on live birth outcomes. The present studies did not compare the efficacy of melatonin with other clinical antioxidants (such as coenzyme Q10, vitamin E) or with any other medication, and did not take into account confounding factors such as age, Body Mass Index (BMI), and metabolic status. Further, the impact of melatonin administration on obstetric complications and follow-up to studies to understand the long-term impact have not been investigated.

## 5. Conclusions

This systematic analysis shows that PCOS patients undergoing ART have higher clinical and chemical pregnancy rates when given melatonin. Melatonin also increases endometrial thickness, the number of mature oocytes, and the proportion of high-quality embryos. Its effectiveness, low cost, and safety make melatonin an attractive adjuvant in ART protocols for PCOS patients. While research has reported varied dosages and durations, 3 mg/day remains the most common and well-tolerated dose. None of the trials included noted any notable side effects. This indicates melatonin is safe for women of reproductive age undergoing ART. In summary, melatonin supplementation improves oocyte maturation, embryo quality, endometrial thickness, and clinical pregnancy rates in women with PCOS undergoing ART. Its safety, affordability, and tolerability make it a promising adjuvant. However, evidence on live birth outcomes, obstetric complications, and long-term effects is limited. Larger, multi-center trials are needed. Future clinical trials may test melatonin’s efficacy compared to other antioxidants and in combination with other drugs. This will help clarify its clinical value. Because PCOS is heterogeneous, future trials should stratify subgroups by phenotypes, androgen levels, OS status, and age. This approach can clarify comparative responses to melatonin in different PCOS subgroups. Trials should also consider circadian rhythm changes in women with PCOS and personalize melatonin administration timing [39]. This will help assess responsiveness, efficacy, and safety. More large-scale, longitudinal studies are warranted to confirm the long-term safety of melatonin for mothers and infants.

## Figures and Tables

**Table 1:** Risk of bias assessment for the studies included in the systematic review.

Study (Author, Year)	Study Design	Risk of Bias	Justification
Mokhtari et al., 2019 [21]	Randomized Clinical Trial	Unclear	Randomization process not clearly described; blinding not reported; outcome data complete.
Pilehvari et al., 2023 [15]	Randomized Clinical Trial	Unclear–Moderate	Limited information on blinding and allocation concealment; small sample size may introduce imprecision.
Kim et al., 2013 [24]	Clinical Study	Moderate–High	Randomization process not clearly defined; lack of blinding; high risk of bias due to deviation from melatonin intervention; low risk of missing outcome data; measurement bias.
Li et al. [3]	Observational Study	High	No melatonin intervention; observational design; high risk of confounding and selection bias.

**Table 2:** Characteristics of included studies in the systematic review.

References	Duration	Country	Sample Size	Inclusion Criteria	Exclusion Criteria
Li et al. [3]	October 2018 to December 2028	China	35 PCOS, 36 non-PCOS	PCOS: Age 25–35 years, PCOS diagnosed by Rotterdam criteria, no oral contraceptives taken for at least 3 months. Non-PCOS: Infertile due to tubal or male factors with normal levels of sex hormones and AMH	Congenital uterine abnormalities, gynecological tumors, endocrine diseases
Pilehvari et al. [15]	2019–2020	Iran	320 PCOS	Age 18–41 years, Rotterdam PCOS criteria, no hormonal medication used in the previous 3 months	Male infertility, tubal infertility, endocrine diseases
Mokhtari et al. [21]	March 2017 to September 2017	Iran	94 PCOS, 100 controls	Aged 20–40 years (28.9 ± 5.5 years), Rotterdam PCOS criteria, normal hysterosalpingography, no underlying endocrine diseases, no hormonal medication use in the previous 3 months	Lack of ovarian response, ovarian hyperstimulation syndrome, no prior infertility treatment history
Kim et al. [24]	2004–2010	Korea	111 PCOS (Phase I) 132 PCOS (Phase II)	PCOS diagnosed using Rotterdam criteria, failed to respond adequately to clomiphene citrate, failure to conceive after multiple cycles of ovulation induction	Not mentioned

PCOS, polycystic ovary syndrome.

**Table 3:** Study design, intervention and ART outcomes in the included studies.

References	Study Design	Procedure	Intervention	Outcome	Conclusion
Li et al. [3]	Observational study	IVF/ICSI	Melatonin measured in follicular fluid on the day of oocyte retrieval	FF melatonin, number of oocytes retrieved, rate of MII oocytes, fertilization rate, rate of top-quality embryos/blastocyst	Lower FF melatonin was found in PCOS compared to controls. The number of oocytes retrieved and IVF rate were significantly increased, and the rate of MII oocytes was higher in the PCOS group; ICSI fertilization rate and high-quality embryos/blastocysts were lower in the PCOS group
Pilehvari et al. [15]	RCT	IVF	3 mg melatonin + 500 mg metformin, administered orally	Number and quality of oocytes/embryos, pregnancy outcome	Improved number of mature oocytes (MII) top-grade embryos, with an increase in implantation and clinical pregnancy rates
Mokhtari et al. [21]	RCT	IUI	3 mg melatonin administered orally on the 3rd day of menstruation until the day of HCG administration	Number of follicles, endometrial thickness, chemical/clinical pregnancy rate	Melatonin improved follicle quality, endometrial thickness, and chemical/clinical pregnancy rates
Kim et al. [24]	Clinical study	IVM-IVF ET	Phase I: Melatonin Supplementation (10 µmol/L) in IVM, with a control group sub-divided into non-stimulated and HCG-primed subgroups. Phase II: HCG priming with melatonin supplementation. Melatonin was added directly to the in vitro culture medium (IVM)	Pregnancy and implantation rates	Melatonin may promote cytoplasmic maturation of immature oocytes and improve pregnancy and implantation rates in PCOS patients in both the non-stimulated and HCG-primed groups

ET, embryo transfer; IVF, in vitro fertilization; ICSI, intra-cytoplasmic sperm injection; IUI, intrauterine insemination; RCT, randomized controlled trial; PCOS, polycystic ovary syndrome; HCG, human chorionic gonadotropin.

## Data Availability

No new data were created or analyzed in this study. Data sharing is not applicable to this article.

## Supplementary Materials

The following supporting information can be downloaded at https://www.mdpi.com/article/10.3390/antiox15070896/s1, Table S1. Search methods for included studies from year 2010–2025 and full texts articles available.

## Abbreviations

AANAT	Arylalkylamine N-acetyltransferase
AMK	N1-acetyl-5-methoxykynuramine
AFMK	N1-acetyl-N2-formyl-5-methoxykynuramine
Akt	Protein Kinase B
Ang-1/2	Angiopoietin-1 and -2
ART	Assisted Reproductive Technology
ASMT	Acetylserotonin O-methyltransferase
BCL-2	B-cell Lymphoma 2
BMI	Body Mass Index
CAT	Catalase
C3OHM	Cyclic 3-hydroxmelatonin
cGCs	Cumulus Granulosa Cells
GLI1	GLI Family Zinc Finger 1
GSH	Glutathione
HAS2	Hyaluronan Synthase 2
HCG	Human Chorionic Gonadotropin
HIOMT	Hydroxyindole-O-methyltransferase
HSP70	Heat Shock Protein 70
IUGR	Intrauterine Growth Restriction
ICSI	Intracytoplasmic Sperm Injection
IUI	Intrauterine Insemination
IVF	In Vitro Fertilization
IVM	In Vitro Maturation
MAPK	Mitogen-activated Protein Kinase
MII	Metaphase II
MT1	Melatonin Membrane Receptor Type 1
MT2	Melatonin Membrane Receptor Type 2
NAS	N-Acetylserotonin
OS	Oxidative Stress
PCOM	Polycystic Ovarian Morphology
PCOS	Polycystic Ovary Syndrome
PMOS	Polyendocrine Metabolic Ovarian Syndrome
PI3K	Phosphoinositide 3-kinase
PKC	Protein Kinase C
PRISMA	Preferred Reporting Items for Systematic Reviews and Meta-Analyses
PTCH1	Patched 1
PTGS1	Prostaglandin-Endoperoxide Synthase 1
PTGS2	Prostaglandin-Endoperoxide Synthase 2
RCT	Randomized Controlled Trial
RNS	Reactive Nitrogen Species
ROS	Reactive Oxygen Species
RORα	Retinoid-related Orphan Receptor α
SHH	Sonic Hedgehog
SMO	Smoothened
VEGF	Vascular Endothelial Growth Factor
VEGFR	Vascular Endothelial Growth Factor Receptor
XIAP	X-linked Inhibitor of Apoptosis Protein
