# Supplementary material for: Effects of Melatonin in Women with Polycystic Ovary Syndrome Undergoing ART: A Systematic Review
Source: Antioxidants (Basel). 2026 Jul 20;15(7):896. doi: 10.3390/antiox15070896 (PMC13403806; doi:10.3390/antiox15070896)
Supplement: Supplementary file 1 [file antioxidants-15-00896-s001.zip › antioxidants-4238791-supplementary.pdf]

Supplemental Table S1. Search methods for included studies from year 2010-2025 and full texts articles available.

Pubmed

#1	"melatonin"[Supplementary Concept] OR "melatonin"[All Fields] OR "melatonin"[MeSH Terms] OR "melatonin s"[All Fields] OR "melatonine"[All Fields] OR "melatonins"[All Fields] OR ("melatonin"[Supplementary Concept] OR "melatonin"[All Fields] OR "melatonina"[All Fields] OR "melatonin"[MeSH Terms]) OR ("melatonin"[Supplementary Concept] OR "melatonin"[All Fields] OR "melatonin"[MeSH Terms] OR "melatonin s"[All Fields] OR "melatonine"[All Fields] OR "melatonins"[All Fields]) OR ("5-methoxy-N-acetyl"[All Fields] AND ("tryptamines"[Supplementary Concept] OR "tryptamines"[All Fields] OR "tryptamine"[All Fields] OR "tryptamine"[Supplementary Concept] OR "tryptamines"[MeSH Terms])) OR ("melatonin/administration and dosage"[MeSH Terms] OR "melatonin/adverse effects"[MeSH Terms] OR "melatonin/deficiency"[MeSH Terms] OR "melatonin/physiology"[MeSH Terms] OR "melatonin/therapeutic use"[MeSH Terms])<br>**Translations**<br>**Melatonin:** "melatonin"[Supplementary Concept] OR "melatonin"[All Fields] OR "melatonin"[MeSH Terms] OR "melatonin's"[All Fields] OR "melatonine"[All Fields] OR "melatonins"[All Fields]<br>**Melatonina:** "melatonin"[Supplementary Concept] OR "melatonin"[All Fields] OR "melatonina"[All Fields] OR "melatonin"[MeSH Terms]<br>**Melatonine:** "melatonin"[Supplementary Concept] OR "melatonin"[All Fields] OR "melatonin"[MeSH Terms] OR "melatonin's"[All Fields] OR "melatonine"[All Fields] OR "melatonins"[All Fields]<br>**tryptamine:** "tryptamines"[Supplementary Concept] OR "tryptamines"[All Fields] OR "tryptamine"[All Fields] OR "tryptamine"[Supplementary Concept] OR "tryptamines"[MeSH Terms]
#2	"in vitro fertilisation"[All Fields] OR "fertilization in vitro"[MeSH Terms] OR ("fertilization"[All Fields] AND "vitro"[All Fields]) OR "fertilization in vitro"[All Fields] OR ("vitro"[All Fields] AND "fertilization"[All Fields]) OR "in vitro fertilization"[All Fields] OR ("reproductive techniques, assisted"[MeSH Terms] OR ("reproductive"[All Fields] AND "techniques"[All Fields] AND "assisted"[All Fields]) OR "assisted

	reproductive techniques"[All Fields] OR ("assisted"[All Fields] AND "reproductive"[All Fields] AND "technology"[All Fields]) OR "assisted reproductive technology"[All Fields]) OR ("fertilization in vitro/drug effects"[MeSH Terms] OR "fertilization in vitro/methods"[MeSH Terms] OR "fertilization in vitro/statistics and numerical data"[MeSH Terms])<br>**Translations**<br>**In Vitro fertilization:** "in vitro fertilisation"[All Fields] OR "fertilization in vitro"[MeSH Terms] OR ("fertilization"[All Fields] AND "vitro"[All Fields]) OR "fertilization in vitro"[All Fields] OR ("vitro"[All Fields] AND "fertilization"[All Fields]) OR "in vitro fertilization"[All Fields]<br>**Assisted reproductive technology:** "reproductive techniques, assisted"[MeSH Terms] OR ("reproductive"[All Fields] AND "techniques"[All Fields] AND "assisted"[All Fields]) OR "assisted reproductive techniques"[All Fields] OR ("assisted"[All Fields] AND "reproductive"[All Fields] AND "technology"[All Fields]) OR "assisted reproductive technology"[All Fields]
#3	"polycystic ovary syndrome"[MeSH Terms] OR ("polycystic"[All Fields] AND "ovary"[All Fields] AND "syndrome"[All Fields]) OR "polycystic ovary syndrome"[All Fields] OR "PCOS"[All Fields] OR ("polycystic ovary syndrome"[MeSH Terms] OR ("polycystic"[All Fields] AND "ovary"[All Fields] AND "syndrome"[All Fields]) OR "polycystic ovary syndrome"[All Fields] OR ("polycystic"[All Fields] AND "ovary"[All Fields] AND "disease"[All Fields]) OR "polycystic ovary disease"[All Fields]) OR ("polycystic ovary syndrome/complications"[MeSH Terms] OR "polycystic ovary syndrome/diagnosis"[MeSH Terms] OR "polycystic ovary syndrome/drug therapy"[MeSH Terms] OR "polycystic ovary syndrome/embryology"[MeSH Terms] OR "polycystic ovary syndrome/history"[MeSH Terms] OR "polycystic ovary syndrome/physiopathology"[MeSH Terms])<br>**Translations**<br>**Polycystic ovary syndrome:** "polycystic ovary syndrome"[MeSH Terms] OR ("polycystic"[All Fields] AND "ovary"[All Fields] AND "syndrome"[All Fields]) OR "polycystic ovary syndrome"[All Fields]<br>**Polycystic ovary disease:** "polycystic ovary syndrome"[MeSH Terms] OR ("polycystic"[All Fields] AND "ovary"[All Fields] AND "syndrome"[All Fields]) OR "polycystic ovary

	syndrome"[All Fields] OR ("polycystic"[All Fields] AND "ovary"[All Fields] AND "disease"[All Fields]) OR "polycystic ovary disease"[All Fields]
	#1 AND #2 AND #3<br>16

Google Scholar

1	Melatonin OR Melatonina OR Melatonine OR 5-methoxy-N-acetyl tryptamine AND Polycystic ovary syndrome OR PCOS OR Polycystic ovary disease AND In Vitro fertilization OR Assisted reproductive technology
	#1 54

Cochrane Library

1	MeSH descriptor: [Melatonin] explode all trees<br>Search: Melatonin OR Melatonina OR Melatonine OR 5-methoxy-N-acetyl tryptamine
2	MeSH descriptor: [Reproductive Techniques, Assisted] explode all trees<br>Search: In Vitro fertilization OR Assisted reproductive technology
3	MeSH descriptor: [Polycystic ovary syndrome] explode all trees<br>Search: Polycystic ovary syndrome OR PCOS OR Polycystic ovary disease
	#1 AND #2 AND #3<br>3

Science Direct

1	Melatonin OR Melatonina OR Melatonine OR 5-methoxy-N-acetyl tryptamine AND Polycystic ovary syndrome OR PCOS OR Polycystic ovary disease AND In Vitro fertilization OR Assisted reproductive technology<br>Advance refine:<br>Year 2010-2025<br>Article type : review, research; Subject area : Biological Science<br>Type: English; Access : Open access
	#1 5005
